# Inflammatory Indices Related to the Postoperative Prognosis of Thymic Epithelial Neoplasms: A Propensity Score Matching Evaluation

**DOI:** 10.1245/s10434-026-19281-1

**Published:** 2026-02-24

**Authors:** Federica Carlea, Alexandro Patirelis, Emanuele Voulaz, Veronica Maria Giudici, Elisa Meacci, Giuseppe Calabrese, Luca Frasca, Pierfilippo Crucitti, Vincenzo Ambrogi

**Affiliations:** 1 Unit of Thoracic Surgery, Department of Surgical Sciences, Tor Vergata University Polyclinic, Rome, Italy; 2 Doctoral School of Microbiology, Immunology, Infectious Diseases and Transplants, MIMIT, Tor Vergata University, Rome, Italy; 3 Department of Thoracic Surgery, IRCCS Humanitas Research Hospital, Milan, Italy; 4 Department of General Thoracic Surgery, Fondazione Policlinico Universitario A. Gemelli-IRCCS, Rome, Italy; 5 Department of Thoracic Surgery, University Campus Bio-Medico, Rome, Italy

**Keywords:** Thymic epithelial tumors, Prognostic factors, Inflammatory Index, Thoracic surgery, Survival

## Abstract

**Background:**

Thymic epithelial tumors presented a variable 5-year survival rate, histological subtype dependent. Efforts have been made to identify new prognostic markers. Neutrophil-to-lymphocyte ratio (NLR), platelet-to-lymphocyte ratio (PLR), and Systemic Inflammatory Index (SII) proved effective in predicting survival in other solid tumors. We evaluated the prognostic significance of NLR, PLR, and SII on disease-free, overall, and tumor-related survivals in patients undergoing surgery with radical intent for thymic epithelial neoplasms.

**Methods:**

We conducted a retrospective analysis in patients operated in four high-volume Italian thoracic surgery centers, followed for a minimum period of 6 months. Immediate preoperative values of NLR, PLR, and SII were recorded. Patients were categorized for each factor based on cutoff values determined statistically by using the Youden index. Survival outcomes were analyzed using Kaplan–Meier curves, log-rank tests, and multivariable Cox regression after propensity score matching (1:1) performed for each inflammatory index.

**Results:**

A total of 376 patients were enrolled. The mean values for NLR, PLR, and SII were 3.0 (3.2), 131.4 (84.1), and 754.6 (28.8), respectively. The calculated cutoff values were 2.9 for NLR, 123.8 for PLR, and 489.0 for SII. After propensity score matching, we obtained 212 cases for NLR, 256 for PLR, and 280 for SII. Multivariable Cox regression analysis revealed a significant association between tumor-related survival and NLR (*p* = 0.030) and SII (*p* = 0.033).

**Conclusions:**

Higher NLR and SII cutoff values can be considered predictors of worse thymic epithelial tumors-related survival after surgery, suggesting their potential role in long-term risk stratification.

**Supplementary Information:**

The online version contains supplementary material available at 10.1245/s10434-026-19281-1.

Thymic epithelial tumors have a variable 5-year survival rate, ranging from 90 to 25%, and a recurrence rate between 3% and 30%.^1^ This is influenced by several factors, including completeness of resection, histological World Health Organization (WHO) classification, and both Masaoka–Koga and TNM staging.^2,3^ However, these variables are often based on postoperative findings and lack preoperative prognostic utility. Given the need for early risk stratification for a tailored management of these patients, it would be desirable to achieve prognostic markers, easily available before the operation and low-cost. Blood serum values have been already proved effective as tumor aggressiveness markers in many other neoplasms.^4–6^ Of many explored markers, the platelet-to-lymphocyte ratio (PLR) and neutrophil-to-lymphocyte ratio (NLR) have been widely investigated as survival predictors in many solid tumors and namely in thymic epithelial tumors.^7–9^ Lately, the Systemic Inflammatory Index (SII) that is neutrophils multiplied for platelets divided by lymphocytes, has been arisen as new promising prognostic marker for thoracic tumors.^10^

The current literature about inflammatory indexes in thymic epithelial tumors is still limited, largely due to the rarity of the disease, which accounts for less than 1% of all tumors.^1^ This relative rarity hinders the building up of prospective studies and favors the retrospective multicentric evaluations.

Our retrospective propensity score matched study aims at evaluating the prognostic significance of NLR, PLR, and SII for disease-free survival (DFS), overall survival (OS), and tumor-related survival (TRS) in a large group of patients undergoing surgery with radical intent for thymic epithelial tumors in many high-volume centers.

## Materials and Methods

### Patient Selection

This retrospective and multicentric study included all patients who underwent surgery with curative intent (implying the entailing of thymus with all visible tumor in the case of nonmyasthenic thymomas and extended thymectomy in the case of myasthenic thymomas) for thymic epithelial tumor from 1997 to 2022 in four major Italian Thoracic Surgery centers: Tor Vergata Polyclinic University (Rome), Agostino Gemelli Foundation Polyclinic University (Rome), Campus BioMedico Polyclinic University (Rome), and Humanitas Clinical Institute (Milan). Preoperative values regarding NLR, PLR, and SII were based on laboratory tests performed during prehospitalization evaluation, always within 2 weeks from surgery. All patients had a minimum follow-up of 6 months. Follow-up was based on phone calls or outpatient visits. Only stage I and II were considered. Exclusion criteria were history of infection or any surgery for any other pathology within the previous 3 months or any malignancy within the past 5 years preceding thymectomy. We also excluded anyone with a positive history of hematological or immunodeficiency diseases. We finally excluded patients with incomplete preoperative laboratory tests and those who failed to attend follow-up within the first 6 months, with the exception of those who experienced a relapse or died. The study was conducted with permission of internal review board (approval n. 143.25). Informed consent for use of personal data for scientific purposes was obtained from all involved subjects.

### Data Collection

Demographics and clinical information were retrieved by clinical records, including gender; age; presence of associated myasthenia gravis and its clinical classification according to Myasthenia Gravis Foundation of America (MGFA); histological classification according to WHO 2014^11,12^; staging according to both Masaoka-Koga classification^13,14^ and TNM according to the eighth edition of the American Joint Committee on Cancer^15^; surgical approach (open vs. minimally invasive, further divided in video-thoracoscopic and robotic surgery); presence of postsurgical residual disease (R1); the use of adjuvant radio- or chemotherapy; preoperative values for NLR, PLR, and SII; follow-up (months); presence of recurrence with its localization (local, regional, or distant); DFS (months); status of the patient (alive or dead); tumor-related death. Disease-free survival was calculated as the period from the day of surgery to first evidence of recurrence, OS as the period from the day of surgery to the death, while TRS as the period from the day of surgery to death due to thymoma.

Once the data had been obtained, a comparative evaluation was made on the fields present in all databases from the four surgical centers. All nonhomogeneous data were resolved, and older cases were restaged according to the last classifications by reviewing each operative and histology report.

### Objectives

The main goal of this study was to evaluate the relevance as prognostic factors for thymic epithelial tumors of inflammatory indexes. In particular, we considered the influence of NLR, PLR, and SII on DFS, OS, and TRS in patients submitted to thymectomy.

### Statistics

Statistical analysis was performed by using the SPSS Statistic program version 26.0 (IBM, Armonk, NY). A *p*-value ≤ 0.050 was considered statistically significant. Categorical variables were reported as whole number and percentage. Continuous variables were presented as mean and standard deviation, given the large number of samples and the normal distribution of the variables. Receiver operating characteristic (ROC) curves and Youden Index^16^ were used to establish cutoff value for inflammatory indexes. Subsequently, a prognostic factor evaluation was made with Kaplan-Meier curves and log-rank test for DFS, OS, and TRS.

Then, a univariable Cox regression analysis was performed by taking this different prognostic factors as covariates: age, dichotomized according to median value (≤59 vs. >59), gender (male vs. female), presence of myasthenia gravis (no vs. yes); surgical approach (minimally invasive vs. open), residual disease (no vs. yes), WHO classification (A-AB-B1 vs. B2-B3), reflecting the evidence that B2 and B3 thymomas usually show a greater invasiveness and worse prognosis compared with A, AB, and B1 ones^17^), TNM staging (I vs. II), adjuvant therapy (no vs. yes), NLR, PLR, and SII (dichotomized according to cutoff emerged from Youden analysis). Masaoka-Koga staging was not included in Cox regression model due to strong correlation with TNM system, thus avoiding multicollinearity and redundancy. Factors significantly influencing survival during univariable analysis underwent multivariable analysis. We included in multivariable analysis only variables resulted statistically significant at univariable analysis in order to minimize the risk of overfitting.

After this preliminary analysis, we used propensity score 1:1 matching as a technical homogenization tool to reduce possible selection bias. A separate matching was performed for each inflammatory marker (NLR, PLR, and SII). For each propensity score matching, the population was divided into two groups (high vs. low) according to the respective cutoff value, and then they were selected and matched one-by-one. We considered the same covariates for each model: age (≤59 vs. >59), gender, presence of myasthenia gravis, surgical approach (minimally invasive vs. open), histological classification (A-AB-B1 vs. B2-B3), and TNM staging (I vs. II stage). Adjuvant therapy and residual disease were not considered among the covariates, because they are postoperative variables usually influenced by pathological and surgical factors already included in the model. Standardized difference less than 0.20 for each covariate was evaluated as acceptable to consider the two groups homogeneous. After propensity score matching, we repeated Kaplan–Meier and Cox regression analysis with the new populations.

## Results

### Demographics and Clinical Characteristics

A total of 376 patients were enrolled in the study. The demographics and clinical characteristics of the patients are summarized in Table 1. The mean age of the patients undergoing surgery was 57.8 (14.8), being 53.2% of female gender. Less than half of the population (43.6%) had myasthenia gravis: 20.7% of them had class I symptoms according to MGFA, 55.5% class II, 19.5% class III, and 4.3% class IV symptoms. Only 36.4% of patients underwent thymectomy with a minimally invasive approach, whereas 63.6% were submitted to open approach. Twenty-five patients (6.6%) showed residual disease postsurgery. According to the WHO classification, 19.9% of patients were classified type A, 21.8% AB, 12.5% B1, 27.4% B2, and 18.3% B3.

**Table 1:** Demographic and clinical characteristics of the patients

*Variable*	
*Age* (*yr*), *mean* (*SD*)	57.8 (14.8)
Gender, *n* (%)	
Male	176 (46.8%)
Female	200 (53.2%)
Center, *n* (%)	
Tor vergata	79 (21.0%)
Gemelli	126 (33.5%)
Campus BioMedico	68 (18.1%)
Humanitas	103 (27.4%)
*Myasthenia gravis, n* (%)	
No	212 (56.4%)
Yes	164 (43.6%)
MGFA class	
I	34 (20.7%)
II	91 (55.5%)
III	32 (19.5%)
IV	7 (4.3%)
Surgical approach, *n* (%)	
Minimally invasive	137 (36.4%)
Open	239 (63.6%)
Residual disease, *n* (%)	
R0	351 (93.4%)
R+	25 (6.6%)
WHO classification, n (%)	
A	75 (19.9%)
AB	82 (21.8%)
B1	47 (12.5%)
B2	103 (27.4%)
B3	69 (18.3%)
TNM stage, *n* (%)	
I	168 (44.7%)
II	208 (55.3%)
Masaoka–Koga stage, *n* (%)	
I	95 (25.3%)
II	73 (19.4%)
III	208 (55.3%)
Adjuvant therapy, *n* (%)	
No	281 (74.7%)
Radiotherapy	93 (24.7%)
Chemotherapy	2 (0.5%)

*MGFA* Myasthenia Gravis Foundation of America; *SD* standard deviation; *WHO* World Health Organization

Regarding the TNM staging, 44.7% of patients belonged to stage I and 55.3% to stage II. After surgery, 74.7% of the population did not undergo any adjuvant therapy, 24.7% received adjuvant radiotherapy, and only 0.5% adjuvant chemotherapy. As regards the inflammatory indices, the mean values were 3.0 (3.2) for NLR, 131.4 (84.1) for PLR, and 754.6 (28.8) for SII.

### Follow-up

The mean follow-up was 58.0 (53.5) months. Regarding the clinical course, 14.4% of patients showed recurrence after surgical treatment. Particularly, 31.5% of them presented local recurrence, 40.7% regional recurrence, and 27.8% distant recurrence. The mean time to relapse was 40.3 (46.3) months. A total of 31/376 (8.2%) of the population died, and 61.3% (19/31) of them died for causes directly correlated to neoplasia growth. The mean postoperative survival was 89.5 (67.7) months, while in the group who died because of the tumor it was 67.2 (65.2) months.

### Cutoff Values for Inflammatory Indexes

The cutoff value for each inflammatory index was found by using the Youden Index, which allows to choose the best value, able to maximize the difference between true positives and false positives, obtained from the ROC curves. This cutoff value was 2.9 for the NLR, 123.8 for the PLR and 489.0 for the SII, respectively. Therefore, patients with values higher than or equal to each cutoff were considered patients with a high preoperative index, whereas subjects with lower values were identified as patients with a low preoperative index.

### Preliminary Analysis

We first evaluated the most impacting factors in terms of DFS, OS, and TRS on the global population (376 patients) with Kaplan-Meier methods and log-rank tests. Within the noninflammatory indexes, DFS was influenced by myasthenia (*p* = 0.031), presence of postsurgical residue (*p* < 0.001), adjuvant therapy (*p* < 0.001), pathological TNM stage (*p* < 0.001), and WHO histological classification (*p* < 0.001). Overall survival was affected only by age (*p* < 0.001) and TNM stage (*p* = 0.003), which also influenced TRS (both *p* < 0.001), together with adjuvant therapy (*p* = 0.029). None of the other factors had a significant impact on any survival.

As far as the inflammatory indexes are concerned, Kaplan-Meier analysis revealed a significant correlation between NLR and DFS (*p* = 0.035) and TRS (*p* = 0.030). Similarly, a higher value of PLR was also correlated with poorer DFS (*p* < 0.001) and TRS (*p* = 0.001). Analogous results were obtained for higher SII for both DFS (*p* = 0.005) and TRS (*p* = 0.030). None of the indexes affected significantly OS.

Afterward, Cox regression analysis was carried out including both noninflammatory and inflammatory prognostic factors. Multivariable analysis demonstrated significant association only for age and stage, showing only a nonsignificant association among any of the three inflammatory indexes and the three survivals.

### Propensity Score Matching Analysis

After preliminary survival analysis, we performed three propensity score matching, each for the specific index (NLR/PLR/SII). The number of pairs obtained was 106 for the NLR index, 128 for the PLR index, and 140 for the SII index for a total of 212, 256, and 280 patients, respectively. Standardized differences before and after propensity score matching were reported in Supplementary Tables S1–S3.

**Fig. 1:**
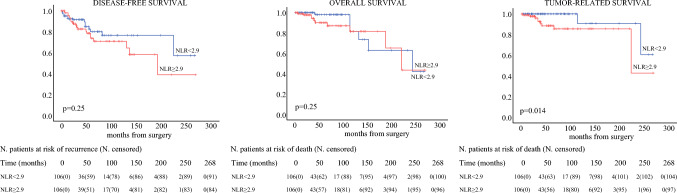
Kaplan-Meier disease-free survival, overall survival, and tumor-related survival curves for neutrophil-to-lymphocyte ratio (NLR) post-propensity score matching

As represented by Kaplan–Meier in Fig. 1, NLR index showed a significant correlation only with TRS (*p* = 0.014) but neither with OS (*p* = 0.25) nor DFS (*p* = 0.25). Similar results were obtained at multivariable Cox regression analysis (Table 2), where a higher value of NLR resulted to be a prognosticator of shorter TRS (hazard ratio [HR] 6.5; 95% confidence interval [CI] 1.2-35.7; *p* = 0.030) together with age and TNM staging.

**Table 2:** Cox regression analysis post-propensity score matching for NLR

	Disease-free survival			Overall survival			Tumor-related survival		
	Univariable	Multivariable		Univariable	Multivariable		Univariable	Multivariable	
	*p*	HR (95% CI)	*p*	*p*	HR (95% CI)	*p*	*p*	HR (95% CI)	*p*
Age (≤59 vs. >59) yr	0.85	–	–	**<0.001**	14.14 (2.91–68.80)	**0.001**	**0.015**	10.36 (1.29–83.17)	**0.028**
Gender (M vs. F)	0.78	–	–	0.68	–	–	0.80	–	–
Myasthenia gravis (no vs. yes)	**0.025**	0.28 (0.13–0.58)	**0.001**	0.55	–	–	0.88	–	–
Surgical approach (minimally invasive vs. open)	0.054	–	**–**	0.57	–	–	0.33	–	–
Residual disease (no vs. yes)	**0.029**	2.12 (0.92–4.92)	0.079	0.69	–	–	0.84	–	–
WHO classification (A/AB/B1 vs. B2/B3)	0.087	–	–	0.44	–	–	0.14	–	–
TNM staging (I vs. II)	**<0.001**	4.03 (2.04–7.90)	**<0.001**	**<0.001**	6.48 (2.02–20.80)	**0.002**	**0.001**	11.96 (2.46–58.06)	**0.002**
Adjuvant treatment (no vs. yes)	**<0.001**	4.63 (2.21–9.70)	**<0.001**	0.41	–	–	0.16	–	–
NLR (<2.9 vs. ≥2.9)	0.25	–	–	0.26	–	–	**0.028**	6.54 (1.20–35.73)	**0.030**

Significant *p*-values are given in bold

*CI* confidence interval; *F* female; *HR* hazard ratio; *M* male; *NLR* neutrophil-to-lymphocyte ratio; *WHO* World Health Organization

**Fig. 2:**
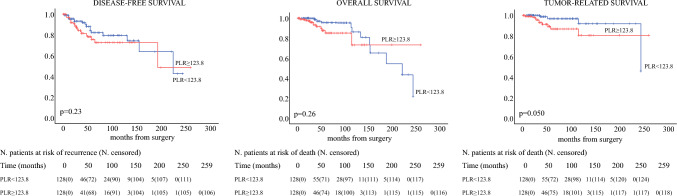
Kaplan-Meier disease-free survival, overall survival, and tumor-related survival curves for platelet-to-lymphocyte ratio (PLR) post-propensity score matching

The postmatching analysis performed for PLR showed significant association of higher index with TRS (*p* = 0.050), but not with OS (*p* = 0.26) nor with DFS (*p* = 0.23) at Kaplan–Meier analysis (Fig. 2). This inflammatory index resulted not affecting any survival at Cox regression analysis, as reported in Table 3.

**Table 3:** Cox regression analysis post-propensity score matching for PLR

	Disease-free survival			Overall survival			Tumor-related survival		
	Univariable	Multivariable		Univariable	Multivariable		Univariable	Multivariable	
	*p*	HR (95% CI)	*p*	*p*	HR (95% CI)	*p*	*p*	HR (95% CI)	*p*
Age (≤59 vs. >59) yr	0.61	–	–	**<0.001**	11.51 (3.28–40.41)	**<0.001**	**0.009**	7.64 (1.69–34.57)	**0.008**
Gender (M vs. F)	0.42	–	–	0.81	–	–	0.76	–	–
Myasthenia Gravis (no vs. yes)	0.27	–	–	0.69	–	–	0.61	–	–
Surgical approach (minimally invasive vs. open)	**0.036**	1.19 (0.42–3.39)	0.74	0.42	–	–	0.18	–	–
Residual disease (no vs. yes)	**0.024**	2.14 (0.93–4.94)	0.074	0.61	–	–	0.47	–	–
WHO classification (A/AB/B1 vs. B2/B3)	**0.008**	0.95 (0.44–2.04)	0.89	0.55	–	–	0.16	–	–
TNM staging (I vs. II)	**<0.001**	3.19 (1.52–6.67)	**0.002**	**0.002**	3.98 (1.72–9.17)	**0.001**	**0.002**	5.61 (1.87–16.82)	**0.002**
Adjuvant treatment (no vs. yes)	**<0.001**	3.04 (1.48–6.25)	**0.002**	0.56	–	–	0.14	–	–
PLR (<123.8 vs. ≥123.8)	0.24	–	–	0.27	–	**–**	0.065	–	–

Significant *p*-values are given in bold

*CI* confidence interval; *F* female; *HR* hazard ratio; M male; *PLR* platelet-to-lymphocyte ratio; *WHO* World Health Organization

**Fig. 3:**
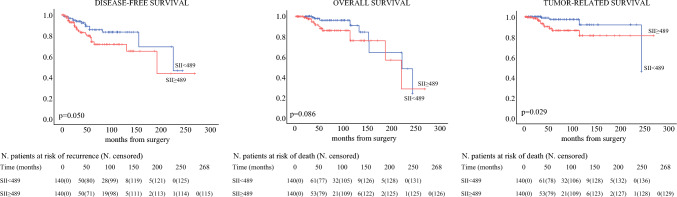
Kaplan-Meier disease-free survival, overall survival, and tumor-related survival curves for systemic inflammatory index (SII) post-propensity score matching

As represented by Kaplan-Meier curves, the postmatching analysis for SII showed a significant association of higher value with both DFS (*p* = 0.050) and TRS (*p* = 0.029) but not with OS (*p* = 0.086) (Fig. 3).

At Cox regression (Table 4), higher SII values were associated to shorter TRS at multivariable analysis (HR 3.6; 95% CI 1.1–11.9; *p* = 0.033) together with age and TNM staging.

**Table 4:** Cox regression analysis post-propensity score matching for SII

	Disease-free survival			Overall survival			Tumor-related survival		
	Univariable	Multivariable		Univariable	Multivariable		Univariable	Multivariable	
	*p*	HR (95% CI)	*p*	*p*	HR (95% CI)	*p*	*p*	HR (95% CI)	*p*
Age (≤59 vs. >59) yr	0.46	–	–	**<0.001**	10.95 (3.07–39.12)	**<0.001**	**0.009**	7.89 (1.76–35.42)	**0.007**
Gender (M vs. F)	0.57	–	–	0.47	–	–	0.33	–	–
Myasthenia gravis (no vs. yes)	0.30	–	–	0.57	–	–	0.75	–	–
Surgical approach (minimally invasive vs. open)	0.14	–	**–**	0.60	–	–	0.19	–	–
Residual disease (no vs. yes)	**<0.001**	3.38 (1.55–7.38)	**0.002**	0.29	–	–	0.31	–	–
WHO classification (A/AB/B1 vs. B2/B3)	**0.017**	0.95 (0.44–2.06)	0.90	0.24	–	–	0.15	–	–
TNM staging (I vs. II)	**<0.001**	3.97 (1.92–9.20)	**<0.001**	**0.001**	4.44 (1.93–10.24)	**<0.001**	**0.001**	7.13 (2.42–20.99)	**<0.001**
Adjuvant treatment (no vs. yes)	**0.001**	1.96 (0.98–3.94)	0.057	0.46	–	–	0.15	–	–
SII (<489.0 vs. ≥489.0)	0.054	–	–	0.094	–	–	**0.040**	3.65 (1.11–11.95)	**0.033**

Significant *p*-values are given in bold

*CI* confidence interval; *F* female; *HR* hazard ratio; *M* male; *SII* systemic inflammatory index; *WHO* World Health Organization.

## Discussion

Our retrospective and multicentric study investigated the pretreatment prognostic value of NLR, PLR, and SII in a large group of patients undergoing surgical resection with curative intent. In literature, the close relationship between inflammation and tumors is well-known.^18^ However, the cause-effect mechanisms related to this interaction are multiple and diversified. Chronic inflammation can initiate tumor growth and promote its progression by interacting with different biological mechanisms.^18^ Alternatively, neoplastic cells can induce inflammatory changes both locally and systemically. Hence, specific inflammatory mediators could be introduced into the systemic circulation with a potential role in metastasis development.^19^

Neutrophils showed an important role in promoting tumorigenesis and metastasis.^20^ Indeed, these cells can release granules containing elastase, collagenase, and gelatinase B; are able to remodel the tumor microenvironment; and accelerate cancer progression.^20–23^ Conversely, tumor cells can directly promote platelets aggregation and activation, thus inducing the release of multiple growth factors able to trigger tumor cell proliferation. Furthermore, lymphocytes demonstrated a key role in the inhibition of tumor proliferation.^24,25^ For these reasons, over the past two decades, several studies have been conducted to analyze the influence of the inflammatory cascade on the long-term survival of cancer patients.

Although a strong correlation between inflammatory indexes and lung cancer has already been demonstrated,^19–22,26^ only in recent years this correlation has been studied in thymic epithelial tumors. In 2021, Huang et al.^27^ established a nomogram to predict outcomes of thymic epithelial tumors by combining clinical characteristics, NLR, PLR, and other blood tests. In this study, the primary outcome was DFS rather than OS. Wang et al.^28^ demonstrated that pretreatment NLR high values were related to increased tumor progression and glycolytic activity in thymic epithelial tumors, but this finding was not associated with survival.

Our study focused on survival of patients who underwent resection of thymic epithelial tumors and took into consideration different inflammatory indexes. To the best of our knowledge, this was one of the first studies to consider TRS. Muriana^29^ and Yanagiya^30^ both demonstrated a correlation between high levels of NLR and a poor prognosis in patients with radical resection of thymic tumors. With reference to high values of SII, Veraar et al. demonstrated a correlation with TRS,^31^ whereas Li et al. showed a significant association with shorter OS.^32^

As regards the PLR, Li et al. recognized this index as significant predictor of shorter prognosis in OS. On the contrary, Sakane et al. did not find any significant correlation of this factor with survival.^33^ These results are concordant with those of the present investigation where PLR did not result a statistically significant prognosticator.

Several limitations related to our study deserve mention. First, it is a retrospective analysis collected from different centers and in a wide timespan, thus presenting potential bias. Furthermore, patients were not made homogeneous in the propensity score with regard to the institution of origin to preserve an adequate number of matched cases for meaningful statistical analysis. However, because all four hospitals are high-volume thoracic centers with great expertise in mediastinal surgery, we do not believe that the institution itself could significantly affect the results. Another important limitation is the relatively short follow-up period. In fact, because of the slow-growth of these tumors with a lower aggressiveness compared with other solid tumors, longer follow-up periods are mandatory to better evidence different prognostic behaviors. Another limitation is represented by the absence in the literature of preestablished limit values for NLR, PLR, and SII. As mentioned, we tried to overcome this by using a widely accepted statistical measure to assess more discriminant threshold values.

Nevertheless, considering the rarity of thymic epithelial tumors, one of the strengths of our analysis is the large sample size. Furthermore, our study was consistent with others but also introduced TRS as an important new endpoint. Indeed, another study with a similar large sample size has already shown a significant correlation of SII and NLR with OS.^32^ However, the association with TRS provided in our investigation might exclude all other possible causes of patient death and makes the association between the inflammatory values and thymic epithelial tumor progression even stronger.

Although our findings suggest that NLR and SII might have a prognostic value, especially in terms of tumor-related survival, it is important to emphasize that our study did not assess whether these markers should influence treatment decisions, such as adjuvant therapy. Further studies would be needed to determine whether elevated inflammatory indices should alter this approach. Nevertheless, these preliminary results suggest that these preoperative markers could serve as an additional tool and improve long-term risk stratification in patients with thymic epithelial tumors.

## Conclusion

Our study demonstrated that higher NLR and SII values could be considered predictors of shorter TRS after surgery. If warranted by further studies, these preoperative markers could become an additional tool, support improved risk stratification, and even contribute to tailor a personalized follow-up in those patients with higher risk of poor prognosis.

## Supplementary Information

Below is the link to the electronic supplementary material.

Supplementary Figure S1 CONSORT diagram showing patient selection process. NLR: neutrophil-to-lymphocyte ratio; PLR: platelet-to-lymphocyte ratio; SII: systemic inflammatory index (JPG 82 kb)

Supplementary file2 (DOCX 17 kb)

Supplementary file3 (DOCX 16 kb)

Supplementary file4 (DOCX 16 kb)
