# Supplementary figures and images for: Inflammatory Indices Related to the Postoperative Prognosis of Thymic Epithelial Neoplasms: A Propensity Score Matching Evaluation
Source: Ann Surg Oncol. 2026 Feb 24;33(6):5368–76. doi: 10.1245/s10434-026-19281-1 (PMC13179239; doi:10.1245/s10434-026-19281-1)

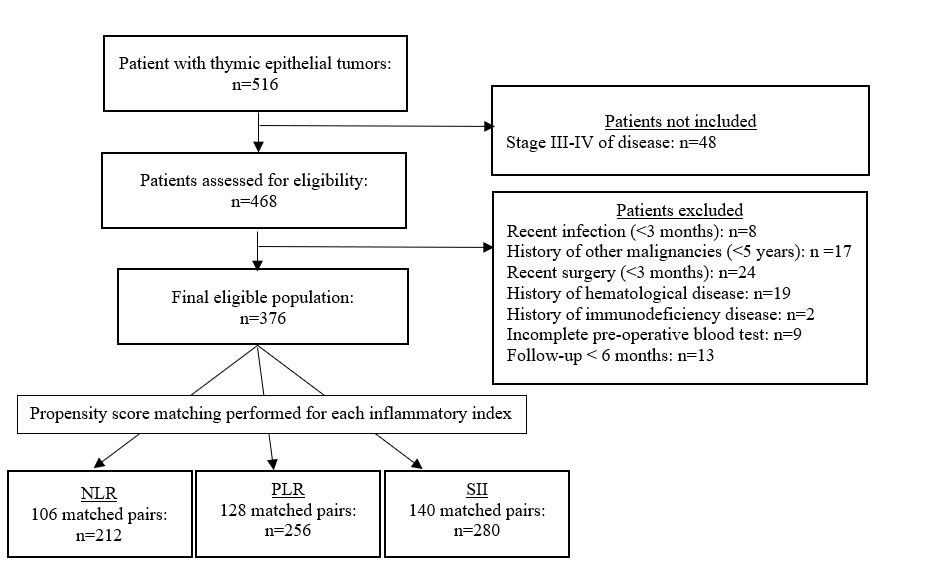

Supplement: Supplementary file 1 — CONSORT diagram showing patient selection process. NLR: neutrophil-to-lymphocyte ratio; PLR: platelet-to-lymphocyte ratio; SII: systemic inflammatory index (JPG 82 kb) [file 10434_2026_19281_MOESM1_ESM.jpg]
