# Supplementary material for: Inflammatory Indices Related to the Postoperative Prognosis of Thymic Epithelial Neoplasms: A Propensity Score Matching Evaluation
Source: Ann Surg Oncol. 2026 Feb 24;33(6):5368–76. doi: 10.1245/s10434-026-19281-1 (PMC13179239; doi:10.1245/s10434-026-19281-1)
Supplement: Supplementary file 2 — Supplementary file2 (DOCX 17 kb) [file 10434_2026_19281_MOESM2_ESM.docx]

|  | **Before propensity score matching** | | | | **After propensity score matching matching** | | | |
| --- | --- | --- | --- | --- | --- | --- | --- | --- |
|  | **NLR<2.9 (n=269)** | **NLR≥2.9 (n=107)** | **p-value** | **Standardized difference** | **NLR<2.9 (n=106)** | **NLR≥2.9 (n=106)** | **p-value** | **Standardized difference** |
| Male gender, n(%) | 119 (44.2) | 57 (53.3) | 0.11 | 0.18 | 57 (53.8) | 56 (52.8) | 0.89 | 0.02 |
| Age>59 years, n(%) | 135 (50.2) | 50 (46.7) | 0.54 | 0.07 | 42 (39.6) | 50 (47.2) | 0.27 | 0.15 |
| Myasthenia Gravis, n(%) | 107 (39.8) | 57 (53.3) | 0.017 | 0.27 | 58 (54.7) | 56 (52.8) | 0.78 | 0.04 |
| Surgical approach, n(%) |  |  | 0.63 | 0.05 |  |  | 1.00 | 0.00 |
| Open | 173 (64.3) | 66 (61.7) |  |  | 65 (61.3) | 65 (61.3) |  |  |
| Minimally invasive | 96 (35.7) | 41 (38.3) |  |  | 41 (38.7) | 41 (38.7) |  |  |
| WHO classification, n(%) |  |  | 0.16 | 0.16 |  |  | 0.89 | 0.02 |
| A, AB, B1 | 152 (56.5) | 52 (48.6) |  |  | 50 (47.2) | 51 (48.1) |  |  |
| B2, B3 | 117 (43.5) | 55 (51.4) |  |  | 56 (52.8) | 55 (51.9) |  |  |
| TNM staging, n(%) |  |  | 0.67 | 0.05 |  |  | 0.89 | 0.02 |
| I | 122 (45.4) | 46 (42.9) |  |  | 47 (44.3) | 46 (43.4) |  |  |
| II | 147 (54.6) | 61 (57.1) |  |  | 59 (55.7) | 60 (56.6) |  |  |

Table S1: standardized difference before and after propensity score matching for NLR. NLR: neutrophil-to-lymphocyte ratio; WHO: World Health Organization
