# Supplementary material for: Inflammatory Indices Related to the Postoperative Prognosis of Thymic Epithelial Neoplasms: A Propensity Score Matching Evaluation
Source: Ann Surg Oncol. 2026 Feb 24;33(6):5368–76. doi: 10.1245/s10434-026-19281-1 (PMC13179239; doi:10.1245/s10434-026-19281-1)
Supplement: Supplementary file 3 — Supplementary file3 (DOCX 16 kb) [file 10434_2026_19281_MOESM3_ESM.docx]

|  | **Before propensity score matching** | | | | **After propensity score matching** | | | |
| --- | --- | --- | --- | --- | --- | --- | --- | --- |
|  | **PLR<123.8 (n=224)** | **PLR≥123.8 (n=152)** | **p-value** | **Standardized difference** | **PLR<123.8 (n=128)** | **PLR≥123.8 (n=128)** | **p-value** | **Standardized difference** |
| Male gender, n(%) | 105 (46.9) | 71 (46.7) | 0.97 | 0.004 | 58 (45.3) | 58 (45.3) | 1.00 | 0.00 |
| Age>59 years, n(%) | 108 (48.2) | 77 (50.7) | 0.64 | 0.05 | 62 (48.4) | 65 (50.8) | 0.71 | 0.04 |
| Myasthenia Gravis, n(%) | 107 (47.8) | 57 (37.5) | 0.049 | 0.21 | 52 (40.6) | 53 (41.4) | 0.90 | 0.02 |
| Surgical approach, n(%) |  |  | 0.93 | 0.008 |  |  | 1.00 | 0.00 |
| Open | 142 (63.4) | 97 (63.8) |  |  | 77 (60.2) | 77 (60.2) |  |  |
| Minimally invasive | 82 (36.6) | 55 (36.2) |  |  | 51 (39.8) | 51 (39.8) |  |  |
| WHO classification, n(%) |  |  | 0.16 | 0.17 |  |  | 0.80 | 0.03 |
| A, AB, B1 | 129 (57.6) | 75 (49.3) |  |  | 69 (53.9) | 71 (55.5) |  |  |
| B2, B3 | 95 (42.4) | 77 (50.7) |  |  | 59 (46.1) | 57 (44.5) |  |  |
| TNM staging, n(%) |  |  | 0.68 | 0.04 |  |  | 0.80 | 0.03 |
| I | 102 (45.5) | 66 (43.4) |  |  | 57 (44.5) | 55 (43.0) |  |  |
| II | 122 (54.5) | 86 (56.6) |  |  | 71 (55.5) | 73 (57.0) |  |  |

Table S2: standardized difference before and after propensity score matching for PLR. PLR: platelet-to-lymphocyte ratio; WHO: World Health Organization
