# Supplementary material for: Inflammatory Indices Related to the Postoperative Prognosis of Thymic Epithelial Neoplasms: A Propensity Score Matching Evaluation
Source: Ann Surg Oncol. 2026 Feb 24;33(6):5368–76. doi: 10.1245/s10434-026-19281-1 (PMC13179239; doi:10.1245/s10434-026-19281-1)
Supplement: Supplementary file 4 — Supplementary file4 (DOCX 16 kb) [file 10434_2026_19281_MOESM4_ESM.docx]

|  | **Before propensity score matching** | | | | **After propensity score matching** | | | |
| --- | --- | --- | --- | --- | --- | --- | --- | --- |
|  | **SII<489 (n=192)** | **SII≥489 (n=184)** | **p-value** | **Standardized difference** | **SII<489 (n=140)** | **SII≥489 (n=140)** | **p-value** | **Standardized difference** |
| Male gender, n(%) | 84 (43.7) | 92 (50.0) | 0.22 | 0.13 | 70 (50.0) | 68 (48.6) | 0.81 | 0.03 |
| Age>59 years, n(%) | 104 (54.2) | 81 (44.0) | 0.049 | 0.20 | 67 (47.8) | 68 (48.6) | 0.90 | 0.02 |
| Myasthenia Gravis, n(%) | 81 (42.2) | 83 (45.1) | 0.57 | 0.06 | 62 (44.3) | 62 (44.3) | 1.00 | 0.00 |
| Surgical approach, n(%) |  |  | 0.66 | 0.04 |  |  | 0.81 | 0.03 |
| Open | 120 (62.5) | 119 (64.7) |  |  | 86 (61.4) | 88 (62.8) |  |  |
| Minimally invasive | 72 (37.5) | 65 (35.3) |  |  | 54 (38.6) | 52 (37.1) |  |  |
| WHO classification, n(%) |  |  | 0.042 | 0.21 |  |  | 1.00 | 0.00 |
| A, AB, B1 | 114 (59.4) | 90 (48.9) |  |  | 74 (52.9) | 74 (52.9) |  |  |
| B2, B3 | 78 (40.6) | 94 (51.1) |  |  | 66 (47.1) | 66 (47.1) |  |  |
| TNM staging, n(%) |  |  | 0.71 | 0.04 |  |  | 0.81 | 0.03 |
| I | 84 (43.7) | 84 (45.7) |  |  | 57 (40.7) | 59 (42.1) |  |  |
| II | 108 (56.3) | 100 (54.3) |  |  | 83 (59.3) | 81 (57.9) |  |  |

Table S3: standardized difference before and after propensity score matching for SII. SII: systemic inflammatory index; WHO: World Health Organization
